# An Exploration of the Relationship Between Gut Virome and Cardiovascular Disease: A Comprehensive Review

**DOI:** 10.31083/RCM36386

**Published:** 2025-06-24

**Authors:** Xiaoyu Wei, Xiaoying Zhang, Mengting Cui, Longrong Huang, Dan Gao, Shengyu Hua

**Affiliations:** ^1^College of Traditional Chinese Medicine, Tianjin University of Traditional Chinese Medicine, 301617 Tianjin, China; ^2^Department of Cardiology, Second Affiliated Hospital of Tianjin University of Traditional Chinese Medicine, 300250 Tianjin, China

**Keywords:** gut virome, bacteriophage, gut microbiota dysbiosis, cardiovascular disease

## Abstract

This study reviews the correlation between the gut virome and cardiovascular diseases (CVDs) and investigates the potential role of the gut virome in CVD progression. The gut virome, which includes bacteriophages and eukaryotic viruses, interacts with the intestinal microbiota and the host immune system, and affects the overall health of the host. Previous studies have demonstrated that alterations in the gut virome are closely associated with various cardiovascular conditions, including hypertension, atherosclerosis, atrial fibrillation, heart failure, and viral myocarditis. Thus, the gut virome may contribute to CVD development by regulating intestinal microecology and immune responses, affecting intestinal barrier function and systemic inflammatory responses. However, despite advancements in gut virome research, our understanding of the specific mechanisms involved and therapeutic potential in CVDs remains limited. Future developments in virus databases and advancements in sequencing technology are expected to offer new insights and methods for the early diagnosis and accurate treatment of CVDs.

## 1. Introduction

The colonization of the human body by microorganisms begins with vertical transmission from the mother during the perinatal period [1]. As the host develops, these microorganisms continue to adapt and evolve, resulting in the establishment of a more stable adult microbiota [2]. Each individual possesses a complex and interconnected array of various microbial communities [3]. Recent studies concur that the symbiotic relationship shared between microbiota and humans is essential in disease onset and resolution [4]. It is not until recently that we find out these microscopic organisms are not ten times [5] but as abundant as human cells [6], with most found in the distal section of the gastrointestinal tract [7].

The intestinal microbiota comprises numerous microorganisms, including bacteria, archaea, fungi, and viruses, with bacteria being the most abundant group [6]. These intestinal microorganisms substantially contribute to the host’s energy metabolism through their metabolites and byproducts [8], in addition to playing a crucial role in developing the host’s immune system [9] to facilitate physiological functions. However, researchers have noted that disruptions in intestinal microbial communities are associated with several disease processes, including neurodegenerative disorders, cardiovascular issues, metabolic conditions, and gastrointestinal diseases [10]. Historically, studies on the association between gut microbial communities and human health or disease conditions have predominantly focused on bacteria. Nonetheless, the emergence of metagenomic high-throughput sequencing technology, bioinformatics analyses, and molecular biology techniques has led to a growing recognition of the intestinal virome’s impact on the human host. Owning to the solution that combines metagenomic sequencing and de novo, several database about human gut virome have been released in the past few years [11, 12, 13], fulfilling the needs for future studies.

The virome found in healthy adult intestines includes bacteriophages and viruses that infect various cellular microorganisms, viruses that affect human cells, and those originating from external environmental sources [14]. Bacteriophages are the most abundant, with an approximate ratio of 1:1 relative to bacteria [15]. The abundant bacterial population within the intestines offers an optimal setting for phage proliferation, while the intestinal microbiota depends on phage predation for its evolutionary progression. This dynamic promotes an antagonistic co-evolutionary interaction between the two [16], indicating that any disturbances in the intestinal microbiota associated with diseases will result in irregular phage populations. Recent studies have predominantly focused on the association between the gut virome and various diseases, especially gastrointestinal disorders, including ulcerative colitis [17], Crohn’s disease [18], colon cancer [19], diabetes [20, 21], and severe acute malnutrition [22], and other metabolic conditions. Recently, there has been a growing interest in revealing possible connections between gut virome and cardiovascular diseases (CVD); however, a significant lack of systematic reviews and comprehensive summaries in this field persists.

CVD includes various conditions affecting the circulatory system, including the heart and blood vessels, such as hypertension, coronary artery disease (CAD), and heart failure. This disease is particularly common among middle-aged and elderly populations. CVD is a major public health issue and remains one of the leading causes of mortality globally [23]. Despite the implementation of comprehensive prevention and treatment strategies in various countries, the global burden of CVD continues to increase, particularly in Asia [24, 25]. The global advancement in life expectancy is projected to decelerate from 2016 to 2040, partly due to the stagnation of improvements in CVD management [25]. Increasing evidence indicates that intestinal microbial derivatives and their metabolites are associated with the onset of CVDs, including atherosclerosis (AS) [26], heart failure (HF) [27], hypertension [28], and atrial fibrillation (AF) [29]. In healthy adults, enteroviruses are predominantly eukaryotic viruses; however, phages dominate most of the gut virome in patients with CVD [30]. This observation highlights the significance of enteroviruses as a crucial component of the intestinal microbiota, highlighting the need for further in-depth study and discussion regarding their role in CVD. Herein, we investigated the relationship between the gut virome and the onset and progression of CVDs and systematically reviewed the latest research advancements related to the gut virome in this context.

## 2. Methods: Literature Search and Synthesis

This study employed a systematic literature review approach, with literature searches covering authoritative databases such as PubMed, Web of Science, and China National Knowledge Infrastructure (CNKI). The core search strategy is demonstrated using PubMed as an example: (“gut virome” OR “bacteriophage” OR “viral microbiome”) AND (“cardiovascular disease” OR “CVD” OR “atherosclerosis”). During the screening process, priority was given to experimental/clinical studies involving the relationship between the gut virome and CVD, as well as the potential role of the gut virome in the development of CVD. The included studies were then comprehensively evaluated and analyzed to understand how the gut virome affects the occurrence and development of cardiovascular diseases by influencing the host’s gut microbiota and immune response.

## 3. Human Gut Virome

Recently, the virome has become a major area of focus in the research on the human intestinal microbiome. Viruses, classified as non-cellular microorganisms, exist within living cells and reproduce through a specific replication mechanism. Historically, humans categorized viruses as “pathogenic agents” that cause diseases, including influenza, acquired immune deficiency syndrome (AIDS), and viral hepatitis. However, due to advancements in high-throughput sequencing technologies, it is increasingly evident that healthy individuals carry a considerable number of viruses, with the gastrointestinal tract frequently exhibiting the highest viral colonization [14]. Enterovirus DNA represents approximately 10% of the total DNA in the intestinal tract, making up approximately 5.8% of the overall DNA of intestinal microorganisms [31]. Additionally, each gram of intestinal content contains approximately 10^9^ virus-like particles (VLPs) [14].

The human gut virome’s composition is complex, marked by its diversity, specificity, and stability. It primarily includes giant, eukaryotic, and prokaryotic viruses and those originating from plants [32]. Camarillo-Guerrero *et al*. [33] analyzed approximately 30,000 human intestinal metagenomes, successfully obtaining >140,000 human enterovirus genomes and approximately 3000 reference genomes of cultured intestinal bacteria. A year-long follow-up study by Shkoporov and Hill [31] on the gut virome of 10 adults revealed that healthy individuals’ gut viromes exhibit a considerable degree of individual variability coupled with relative stability. Nishijima *et al*. [34] examined over 4000 human gut viromes and identified 97 factors affecting the virome structure, which included age, gender, and diet. Diseases and medications have the most substantial influence on the virome. The virome in the intestine interacts with the intestinal microbiota and host immunity, playing a crucial role in human health and disease.

### 3.1 Gut Virome and Intestinal Microecology

The intestines host a diverse array of microorganisms, including prokaryotic bacteria, eukaryotic fungi, archaea, protozoa, and viruses. Bacteriophages are the most abundant viral forms present in the human gut virome within this diverse ecosystem [35]. These viral agents possess a simple structure, primarily comprising capsid proteins and nucleic acids. Bacteriophages adhere to specific receptors on the surface of host cells through their capsid proteins, subsequently delivering their nucleic acids into the cells. They progress through several phases, which include nucleic acid replication, protein synthesis, and progeny assembly within the host cells, thus facilitating their proliferation [36]. The interplay between enteroviruses, intestinal bacteria, and intestinal fungi is crucial in maintaining intestinal homeostasis and significantly affects human health and disease.

#### 3.1.1 Gut Virome and Intestinal Bacteria

Phages in the gut virome directly affect the composition, quantity, and characteristics of host bacteria [14] and create cascading effects on other bacterial communities by modifying their interactions. This regulation affects bacterial metabolism and viability, subsequently influencing various metabolites, including amino acids, peptides, carbohydrates, lipids, and nucleotides [37]. A previous study has demonstrated that the Bacteroidetes phage BV01 modifies the transcriptional activities of host genes, thereby affecting bile acid metabolism [38]. Bile acids and their secondary metabolites produced by intestinal microorganisms are closely associated with conditions such as hypertension, AS, arrhythmia, heart failure, and several metabolic disorders [39, 40]. Consequently, phage colonization induces alterations in intestinal bacterial composition and metabolism, factors essential for host health. Furthermore, phages are instrumental in facilitating gene transfer across bacterial communities. Phages can transfer DNA between bacterial cells, which can introduce new functions into their genomes and may impact bacterial adaptability and virulence [41, 42]. Additionally, horizontal gene transfer mediated by phages can enhance the stability of these bacterial communities, potentially clarifying the long-term involvement of the gut virome in managing certain diseases [43]. Moreover, the relationship between phages and bacteria extends beyond direct defensive and counter-defense interactions; it includes genetic exchange and mutations, thus playing a key role in their co-evolution. This ongoing coexistence exemplifies a continuous co-evolutionary balance, fundamentally stemming from the persistent adaptation and conflict between both entities. In the intestinal environment, phages and bacteria are mutually dependent and exist in a fluctuating equilibrium [44], where any disruption could result in disease.

#### 3.1.2 Gut Virome and Intestinal Fungi

Besides bacteria and viruses, the human gastrointestinal tract harbors fungal communities, constituting approximately 0.1% of the overall microbial population in the intestines. These fungi are essential for maintaining intestinal homeostasis and can influence the onset and progression of various diseases. A previous study has demonstrated that intestinal fungi might influence the infection and replication mechanisms of enteroviruses by modifying the intestinal environment [45]. For instance, the presence of these fungi can modify the immune response in intestinal epithelial cells, subsequently impacting the infectivity of enteroviruses [46]. This complex interaction compels us to investigate the effects of fungi and viruses in studies related to intestinal health and diseases. Investigating the association between intestinal fungi and enteroviruses is a significant yet somewhat overlooked field of research. Gaining a more profound understanding of this relationship may shed light on further disease mechanisms and pave the way for innovative treatment approaches in the future.

### 3.2 Gut Virome and Host Immunity

#### 3.2.1 Gut Virome and Intestinal Mucosal Barrier

The intestinal mucus layer primarily comprises mucins that are heavily glycosylated and secreted by goblet cells in the intestine. The small intestine possesses a single layer of mucus gel, while the colon comprises two separate layers: an outer loose layer inhabited by commensal bacteria and an inner dense layer that is sterile. This intestinal mucus barrier comprises the mucus layer, antimicrobial peptides (AMPs) [47] secreted by Paneth cells located at the base of the epithelial crypts in the small intestine, and secreted immunoglobulin A (sIgA) [48]. This barrier serves as the primary physical defense within the intestine, effectively preventing direct interactions between pathogens and epithelial cells [49]. A previous study has demonstrated a significant prevalence of phages within the mucus barrier, with their concentration in the mucosal layer being 4.4 times higher than that in the surrounding non-mucosal area [50]. Phages can adhere to the intestinal mucus by interacting with glycan residues in mucus proteins through the immunoglobulin-like domain of their capsid proteins [51]. Under conditions of low to moderate mucin levels, the T4 phage can momentarily bind to mucin using the Hoc protein, which decelerates its movement and promotes sub-diffusion within the mucus layer. This movement enhances the T4 phage’s ability to efficiently locate adhering bacterial hosts within the local mucus network, subsequently increasing the likelihood of interactions between phages and bacterial hosts while strengthening the antibacterial response [52]. Therefore, the mucus barrier is essential for protecting intestinal epithelial cells from enterovirus attacks and facilitating interactions between phages and bacteria.

#### 3.2.2 Gut Virome and Intestinal Epithelial Barrier

Intestinal epithelial cells are essential for the selective absorption of nutrients and for establishing an intestinal epithelial barrier that is crucial for regulating interactions between pathogenic microorganisms and immune cells within the intestine. Previous studies demonstrated that the gut virome may engage directly with intestinal epithelial cells, which are equipped with pattern recognition receptors (PRRs) that enable the detection of enteroviral particles. Additionally, the gut virome may interact with the epithelial barrier indirectly, with intraepithelial lymphocytes (IELs) within the intestinal mucosa playing a crucial role in gut immune defense. The abundance of external antigens in the intestine allows IELs to perform significant immune functions while exhibiting immunosuppressive characteristics, thereby protecting the intestinal epithelial barrier from damage due to excessive immune responses [53]. Besides, it is widely recognized that enteroviruses predominantly infiltrate the human body when the intestinal epithelium is compromised or its permeability is altered; however, experimental evidence reveals that phages can cross the epithelial barrier even under normal physiological conditions [54]. For instance, *Escherichia coli* phages administered orally can traverse epithelial barriers and migrate to remote organs, including the spleen, thereby triggering immune responses [55]. Research has uncovered two primary mechanisms through which phages can penetrate the intestinal epithelium: through internalization using the endocytic pathway and through transcytosis. Besides these two methods, while the Trojan horse theory remains to be conclusively validated, there is a possibility that phages may infiltrate epithelial cells by targeting bacteria, thus aiding in the crossing of the epithelial barrier [56]. The gut virome interacts with the intestinal epithelium directly and indirectly and can navigate through the epithelial barrier to exert regulatory influences on distant organs. This may be the key to the role of the gut virome in CVD development.

#### 3.2.3 Gut Virome and Immune Cells

Bacteriophages comprise the largest viral community in the human gut virome, while eukaryotic viruses are essential in the onset of diseases in the body. Different types of viruses engage with the immune system through various mechanisms. Phages affect the intestinal bacterial population by integrating genes and causing bacterial lysis, which allows them to indirectly modulate human immune responses [57]. Furthermore, when they penetrate the intestinal epithelial barrier, phages may engage with dendritic cells (DCs) and macrophages, activating humoral immunity and thus having a direct effect on the immune system. Miedzybrodzki *et al*. [58] reported that phages can reduce reactive oxygen species (ROS) population during infections from Gram-negative bacteria by interacting with neutrophils and lipopolysaccharide (LPS) and lysing the bacteria, which lessens organ damage. Similarly, Miernikiewicz *et al*. [59] reported that the bacteriophage T4 tail synthase gp12 can modify the structure of LPS by binding to it, thereby affecting the inflammatory response triggered by LPS. Hence, phages can affect inflammation [59]. However, it is crucial to recognize that phages can produce pro-inflammatory responses. In the course of bacterial infections, phages produced after infecting harmful bacteria can be taken up by immune cells, which activate antiviral responses that hinder bacterial clearance and worsen the infection [60]. Gogokhia *et al*. [61] reported that phage capsid proteins and DNA may prompt DCs to release IFN-γ, consequently aggravating colon inflammation.

Similar to gut microbiota, eukaryotic viral infections produce beneficial and detrimental effects on the host. Research associated with noroviruses has demonstrated that in germ-free or antibiotic-treated mice, MNV-CR6 can reverse immune deficiency through type I interferon (IFN-1)-dependent development of lymphocytes [62]. However, MNV-CR6 leads to distorted morphology and impaired function of Paneth cells in Atg16L1HM mice, resulting in reduced immune function [63]. Interleukin-22 (IL-22), predominantly generated by type 3 innate lymphocytes (ILC-3) within the intestine, is essential for intestinal tissue repair and the modulation of intestinal microbiota [64]. Research suggests that the activation of the ILC-3 and IL-22 pathways is a key element in how murine norovirus (MNV) enhances intestinal repair and increases survival rates in 3-week-old wild-type mice infected with the Gram-negative bacterium *Citrobacter rodentium* [65]. Upon viral infection, the nucleic acid is identified by the intrinsic PRRs located on intestinal epithelial cells and innate immune cells, prompting these immune cells to generate inflammatory mediators, including interferon, interleukin, and tumor necrosis factor, to eliminate the virus, leading to intestinal inflammation. Conversely, studies indicate that in wild-type mice infected with mouse cytomegalovirus (MCMV), IFN-I can transmit signals through macrophages, which stimulates the proliferation of epithelial cells across multiple organs, including the colon [66]. Furthermore, T cells and B cells are crucial in viral infections [67, 68]. Research indicates that mice devoid of T cells and B cells exhibit increased vulnerability to chronic rotavirus infections. During acute MNV infection, CD8+ T cells exhibit enhanced capabilities for virus clearance, thereby facilitating more effective recovery from infection compared to what is observed in chronic MNV infection [69].

The interaction between enteroviruses and immune cells is complex and multifaceted. This relationship resembles the symbiotic interactions between bacteria and the human body.

## 4. Gut Virome and CVD

Recent research increasingly demonstrates that the human gut virome is significantly associated with human health and various diseases. Its impact goes beyond inflammatory bowel disease (IBD) [70] and irritable bowel syndrome (IBS) [71], encompassing conditions such as diabetes mellitus (DM) [72], alcohol-related liver disease (ALD) [73], and additional health issues. Furthermore, the gut virome’s involvement in CVDs, including hypertension, AS, AF, heart failure, and viral myocarditis, has been recognized [74], highlighting the need for increased focus.

### 4.1 Hypertension

Hypertension is a cardiovascular disease characterized by the persistent elevation of systemic arterial blood pressure. This syndrome presents a considerable risk for a range of cardio-cerebral diseases, including stroke, CAD, and HF. According to the first “Global Hypertension Report” published by the World Health Organization in 2023, a study from 2019 demonstrated that 33% of individuals aged 30–79 were affected by hypertension globally (which is categorized as a systolic blood pressure (SBP) of ≥140 mmHg, a diastolic blood pressure (DBP) of ≥90 mmHg, or those receiving medication). Annually, approximately 10 million people die of high systolic blood pressure [75]. The onset of hypertension is mainly linked to a combination of genetic predispositions [76] and environmental factors, including salt consumption [77], obesity [78], stress [79], and the intake of alcohol [80]. However, the precise origins of hypertension have yet to be fully identified. Recently, a growing body of research has demonstrated a significant association between the emergence of hypertension and gut microorganisms [81]. Although the interactions between gut bacteria, their metabolites, and hypertension have been thoroughly investigated, the relationship between the gut virome and hypertension remains under-researched.

A previous study demonstrated that the rate of isolation for enteroviruses in patients with hypertension and hypertensive heart failure (HHF) was significantly greater than what was seen in the group with normal blood pressure. Among the enteroviruses identified, echoviruses, along with coxsackievirus B5, exhibited the most significant isolation rates [82]. In addition, a separate study encompassing 1030 participants found an association between coxsackievirus and hypertension [83]. As a result, the potential association between coxsackievirus, categorized as an enterovirus, and hypertension calls for additional research. Moreover, enterovirus A71 has been linked to hypertension. A retrospective analysis involving patients with severe hand, foot, and mouth disease demonstrated that 15% of those patients developed hypertension [84]. This occurrence could be associated with the systemic inflammatory response and acute cardiovascular injury triggered by enterovirus A71 [85].

Recently, substantial findings regarding the gut virome in patients with hypertension have emerged due to advancements in metagenomic high-throughput sequencing technology. A comprehensive cohort study conducted in Japan demonstrated the association between the gut virome and 1941 patients with hypertension [34]. Han *et al*. [86] made extensive use of publicly available data resources to carefully analyze 196 fecal metagenomic datasets associated with hypertension, uncovering that enteroviruses are more responsive than bacteria for diagnosing hypertension at an early stage. The viruses identified during this investigation included *Streptococcus* virus phiAbc2, Cronobacter phage CR3, and *C. medinalis* granulovirus, potentially affecting the onset of hypertension [86]. Additionally, a correlation analysis between blood pressure and the gut virome conducted by Ye HL *et al*. [87] revealed that Pagevirus was positively related to SBP and DBP, while associations were observed between Mimivirus and Deltaentomopoxvirus with SBP. Furthermore, the Betterkatz virus exhibited a positive correlation with DBP, and the Taranis virus exhibited a negative correlation with SBP. The researchers discovered a tandem relationship between the oral and gut virome concerning hypertension [87]. Furthermore, experiments with animals indicated that the enterovirus group of mice with Ang II-induced hypertension revealed non-significant differences in comparison to the control group. A diet rich in fiber could alleviate Ang II-induced hypertension through intricate interactions with intestinal bacteria and phages [88], thus deepening our understanding of the interplay between diet, phages, and hypertension (Table 1, Ref. [34, 82, 83, 84, 86, 87, 88]).

**Table 1.** **Research works investigating the relationship between the gut virome and hypertension**.

Disease/Model	Subjects	Determination	Main Findings	References
Hypertension, dilated cardiomyopathy (DCM), HHF/humans	70 personal stool specimens (65 patients and 5 controls)	Hypertension, DCM, HHF, and enteroviruses correlations	Patients with hypertension, HHF, and DCM exhibited a higher rate of isolation for enteroviruses, especially Coxsackie-B5-viruses and echoviruses, than the controls.	[82]
Hypertension/humans	1300 Chinese Mongolians aged ≥30 (488 patients with hypertension and 942 normotensive participants)	Using enzyme-linked immunosorbent assay to detect IgG antibodies for Coxsackie viruses	Significant association between seroprevalence of Coxsackie virus and hypertension.	[83]
Hypertension caused by hand, foot, and mouth disease (HFMD)/humans	147 severe patients with HFMD	Patients’ data were analyzed and presented as mean ± standard deviation or percentages	15.5% of the 147 severe patients with HFMD had hypertension.	[84]
Hypertension/humans	4198 Japanese personal fecal samples	Metagenomic sequencing data of fecal samples	1941 individuals exhibited a significant association between hypertension and gut virome.	[34]
Hypertension/humans	196 fecal samples	Identification of the viral and bacterial composition of fecal samples	-For hypertension in the early stage, enteroviruses are more responsive than bacteria in diagnosis.	[86]
			-*Streptococcus* virus phiAbc2, Cronobacter phage CR3, and *C. medinalis* granulovirus can affect the onset of hypertension.	
Hypertension/humans	180 samples, including feces, subgingival plaques, and saliva	Metagenomic sequencing of viruses and bacteria of oral and fecal samples	-Pagevirus was positively related to SBP and DBP.	[87]
			-Mimivirus and Deltaentomopoxvirus were positively related to SBP. Betterkatz virus exhibited a positive correlation with DBP.	
			-Taranisvirus exhibited a negative correlation with SBP.	
			-A Tandem between oral and gut virome in hypertension was discovered.	
Hypertension/mice	4 male C57BL/6J mice per group were implanted with minipumps containing saline or angiotensin II, and 4 male C57BL/6J mice per group with a diet rich in resistant starches or a diet lacking resistant starches	Characterization of bacterial and viral genomes, sequencing bulk and VLPs dsDNA	-Enterovirus group of mice with Ang II-induced hypertension exhibited non-significant differences in comparison to the control group.	[88]
			-Diets rich in fiber could alleviate Ang II-induced hypertension through complex interactions with intestinal bacteria and phages.	

HHF, hypertensive heart failure; SBP, systolic blood pressure; DBP, diastolic blood pressure; VLPs, virus-like particles.

### 4.2 AS and Coronary Heart Disease (CHD)

AS is a common disease that significantly threatens human health. One form of AS is CAD. A significant feature of AS lesions is the deposition of lipids beneath the intima in particular areas of the artery, which is associated with the proliferation of smooth muscle cells and components of the fibrous matrix, leading to the formation of atherosclerotic plaques [89]. When AS affects the coronary arteries, it may result in complete blockage of the vessel and subsequent myocardial infarction; similarly, if it occurs in the blood vessels of the brain, such obstructions may cause cerebral infarction, often referred to as stroke. This condition constitutes the fundamental pathological basis for ischemic diseases affecting the cardiovascular and cerebrovascular systems [90]. The mechanisms behind AS are complex and multifaceted. Previous studies have demonstrated that the theories related to its pathogenesis predominantly include inflammation [91], lipid accumulation [92], oxidative stress [93], and damage to the endothelium, among other factors. However, there is no singular theory that can completely explain the pathogenesis of AS. Apart from the known risk factors such as hypertension, diabetes, hypercholesterolemia, and smoking, other elements, including life stress and environmental factors, can significantly contribute [94]. Data indicates that AS is the major cause of death and illness among patients with various cardiovascular diseases globally, with deaths related to AS comprising approximately 50% of cardiovascular disease-related deaths in Western nations [95]. Thus, investigating the underlying mechanisms and potential treatments for AS is crucial.

A significant relationship exists between viral infections and AS [96]. The infection caused by human cytomegalovirus (HCMV) is considered a major factor in the development of AS [97]. HCMV is a double-stranded DNA virus that is classified within the beta subfamily of herpesviruses. This virus can infect various organs, including the intestines [98]. Numerous studies have demonstrated a correlation between HCMV seropositivity and an increased risk of coronary AS [99, 100]. These studies have indicated the association between viral infections and AS.

Recently, driven by developments in high-throughput metagenomic sequencing technologies, there has been a growing focus in research on the association between CHD and the gut virome. Clinical case-control investigations have demonstrated that the gut virome of patients with CHD significantly contrasts with that found in healthy individuals. One investigation highlighted that Siphoviridae was significantly more prevalent in the virome of patients with CHD [101], while another identified Virgaviridae and Microviridae as the major types of viruses in those with CHD [102]. A substantial cohort study in China analyzed variations in phage populations, demonstrating that several phages exhibited increased abundance or reduction in the gut microbiomes of patients with CHD compared to healthy controls. The recognized hosts of phages associated with CHD primarily comprised bacteria from the *Enterobacteriaceae* and *Streptococcus* families [26]. Additionally, recent studies have demonstrated that Tsarbombavirus is a potentially valuable phage regarding CHD. It can affect the microbiota and metabolic pathways associated with CHD. Among the involved metabolites, sn-glycero-3-phosphocholine and glycerophosphocholine are recognized as key differential metabolites about lipid metabolism in CHD, demonstrating a robust positive relationship with Tsarbombavirus [103].

Studies on this subject are scarce, making it challenging to ascertain the consistent direction of gut virome changes in CHD; these findings strongly suggest that changes in the gut virome are closely associated with the onset and progression of AS and CHD (Table 2, Ref. [26, 101, 102, 103]). Considerable work remains to be done in the future.

**Table 2.** **Research works investigating the relationship between gut virome and AS and CHD**.

Indication/Model	Subjects	Determination	Main Findings	Reference
ACVD/humans	214 patients and 171 controls	Identify viral operational taxonomic unit (vOTU) and analyze changes in their abundance	Significant increase in viral richness and a visible alteration in virome structure.	[101]
CHD/humans	37 patients and 6 controls	Viral metagenomic investigation of fecal samples	-Virgaviridae and Microviridae were the two dominant viruses found in the enteric virome of patients with CHD.	[102]
ACVD/humans	218 patients and 187 controls	Viral and bacterial metagenomic investigation of fecal samples	The gut microbiota of patients with ACVD exhibited a higher abundance of *Enterobacteriaceae* and *Streptococcus* spp.	[26]
CHD/mice	15 mice in the CHD group and 15 mice in the sham group	Enteroviral metagenomics and serum UPLC–MS/MS metabolomics	- 24 different species of the virus have been found.	[103]
			- Tsarbombavirus may be associated with the dominant genus of CHD-associated metabolites.	

AS, atherosclerosis; CHD, coronary heart disease; ACVD, atherosclerotic cardiovascular disease.

### 4.3 AF

AF is a common sustained arrhythmia that frequently arises from the remodeling of the atria due to various cardiac conditions. This disorder substantially increases the risk of death, stroke, heart failure, cognitive impairment, and dementia, which negatively affects the quality of life of patients [104]. The incidence of AF increases with age, and as the global population ages, it is anticipated that AF will considerably strain healthcare systems and society [105]. Despite progress in AF ablation technology providing more treatment alternatives, a previous study has demonstrated that approximately 50% of those affected still need repeat procedures [106]. The mechanisms behind AF are unclear, highlighting the need for additional research into the fundamental processes associated with this condition.

Numerous studies have demonstrated that gut microorganisms substantially influence human health and disease. Approximately 40% of patients with AF report gastrointestinal issues, including indigestion [107]. Accordingly, there is a growing need to focus on intestinal microecology within AF studies. The complex interplay between gut microbiota and AF has received considerable agreement within the scientific community [108]. However, studies examining the association between enteroviruses and AF are still in their early phases. A recent case-control study discovered notable shifts in enteroviral composition and diversity among patients with AF [109]. Specifically, there was a substantial increase in the levels of *Streptococcus* virus DT1 and Pseudomonas phage in those with AF, while the association of Synechococcus phage S-SM1 and Cronobacter phage CR5 with bacterial species was significantly reduced. Hence, the researchers formulated a risk prediction model, indicating that the gut virome could be valuable for screening and stratifying individuals at high risk for latent AF (Table 3, Ref. [109]). In this regard, Li N *et al*. [110] proposed the idea of a gut-immune-cardiac axis, indicating that gut microbiota might increase AF risk by affecting the immune system, thereby indirectly leading to AF development. This mechanism could serve as a pathway through which the gut virome promotes AF.

**Table 3.** **Research works investigating the relationship between gut virome and AF**.

Indication/Model	Subjects	Determination	Main Findings	Reference
AF/humans	50 patients and 50 controls	Combined with metagenomic data, enterovirus signaling was analyzed	- The associations of Synechococcus phage S−SM1 and Cronobacter phage CR5 with bacterial species were tight but dampened in AF	[109]
			- The gut viral signatures are associated with AF.	

AF, atrial fibrillation.

### 4.4 HF

HF is a clinical condition predominantly caused by structural and/or functional abnormalities within the heart. This syndrome results in elevated intracardiac pressure and/or insufficient cardiac output at rest or during physical activity, presenting various clinical symptoms [111]. Additionally, HF signifies the advanced stage of CVDs, including hypertension, and correlates with the decompensation phase of cardiac insufficiency. For many years, researchers have investigated the association between the development of HF and changes in intestinal microbiota [112], although a clear causal link has not yet been confirmed [113].

Ischemia and hypoxia are frequently associated with heart failure. Decreased blood flow to the intestines results in intestinal ischemia, swelling, and inflammation. This sequence of events undermines the intestinal barrier function and disturbs the balance of intestinal microorganisms, enabling these microorganisms and their metabolites to enter the bloodstream. Pathogenic bacteria and viruses can trigger inflammatory responses, while trimethylamine N-oxide found in their byproducts is essential in the development and progression of conditions such as AS and HF. However, short-chain fatty acids in these metabolites demonstrate protective properties against inflammation and vascular injury [114]. A previous study has demonstrated that patients with chronic HF exhibit a marked increase in pathogenic bacteria and *Candida* proliferation within their intestines as their health declines, particularly when compared to healthy individuals [27]. However, the connection between enteroviruses, another key element of gut microbiota, and heart failure has not been thoroughly investigated. Recent studies have revealed that the levels of enterococcal phages change in cases of irreversible right heart failure caused by pulmonary arterial hypertension (PAH) and that this alteration is associated with the levels of enterococci present (Table 4, Ref. [115]). The involvement of enteroviruses in heart failure development and progression is unclear and deserves additional research.

**Table 4.** **Research works investigating the relationship between gut virome and HF**.

Disease/Model	Subjects	Determination	Main Findings	References
PAH/humans	Stool specimens of 18 patients with type 1 PAH and 13 controls	Shotgun metagenomic analyses of viromes of stool specimens	Lactococcal phages were relatively decreased while Enterococcal was enriched.	[115]

HF, heart failure; PAH, pulmonary arterial hypertension.

### 4.5 Viral Myocarditis

Myocarditis is an inflammatory condition that can be localized or widespread, impacting cardiac tissue. Symptoms can range from mild manifestations, including chest discomfort and slight dyspnea, to severe situations that may result in acute cardiogenic shock [116]. The primary trigger for myocarditis is viral infection [117], with coxsackievirus B3 (CVB3) responsible for approximately 25% of viral myocarditis cases among enteroviruses [118]. In the gastrointestinal tract, coxsackievirus works with adenovirus to invade host cells by leveraging the coxsackie and adenovirus receptor (CAR) [119], which activates systemic viremia and promotes an inflammatory response in the vasculature. The virus is subsequently spread to the heart through systemic circulation, where it attaches to receptors on cardiomyocytes. After uncoating, the virus replicates inside these myocardial cells. Considering CVB3 specifically, its viral protease 2A causes damage, apoptosis, and cardiomyocyte necrosis [120]. This initial stage marks the activation of the innate immune response in reaction to the virus. In the following stage, the adaptive immune response takes precedence. The immune system identifies the infected myocardial cells, resulting in the infiltration of macrophages, NK cells, and T cells, which specifically attack and destroy the infected myocardial cells. These immune cells release significant amounts of cytokines, worsening myocardial injury and leading to cardiac systolic dysfunction. In the later phases, as the viral load diminishes, most patients may see a restoration of cardiac systolic function; however, some patients may not fully regain myocardial function and can develop a chronic condition [117]. Recent studies demonstrated that CVB3 infection may alter intestinal microbiota and its metabolic balance, potentially providing new insights into the pathogenesis of acute viral myocarditis (AVMC) (Table 5, Ref. [121]).

**Table 5.** **Research works investigating the relationship between gut virome and viral myocarditis**.

Disease/Model	Subjects	Determination	Main Findings	References
AVMC/mice	20 pathogen-free male BALB/c mice	Determination of GM composition and metabolites in the colon by 16S rRNA gene sequencing and ultra-high-performance liquid chromatography-tandem mass spectrometry (UHPLC-MS/MS) untargeted metabolomics profiling	CVB3 infection may alter intestinal microbiota and its metabolic balance.	[121]

AVMC, acute viral myocarditis; CVB3, coxsackievirus B3; GM, gut microbiome; BALB/c, Baggalb/c.

## 5. Discussion

### 5.1 Possible Mechanisms of Gut Virome and CVD

The gut virome, a collection of enteroviruses in the gastrointestinal tract, is implicated in the pathogenesis of CVDs, particularly through its interactions with the immune system and its effects on cardiac tissues. For instance, CVB is a well-studied pathogen associated with myocarditis, and it evades the host’s immune response, particularly by modulating type I IFN signaling pathways. The type I IFN response is essential for controlling viral infections, and enteroviruses have evolved various strategies to antagonize this pathway, facilitating their replication and persistence in cardiac tissues [122]. In CVB, studies have demonstrated that specific viral RNA forms, including the 5^′^-terminally deleted variants, can impair IFN-β production in cardiomyocytes [123]. This impairment leads to a reduced antiviral response, which permits the virus to replicate more effectively and contributes to myocarditis development [123]. These viral forms in the heart can lead to chronic inflammation and damage, key factors in the progression of dilated cardiomyopathy. Furthermore, enteroviruses can manipulate host cellular processes to enhance their replication. For instance, they can hijack cellular machinery and pathways, including the mitochondrial antiviral signaling pathway, to evade apoptosis and prolong their replication cycle. This manipulation can lead to cellular stress and damage, further contributing to cardiac dysfunction [122, 124]. The interaction between the gut virome and the host immune system is complex, as these viruses can induce pro-inflammatory and anti-inflammatory responses, which may influence the severity of cardiac injury. Furthermore, the role of the gut virome in CVD is not limited to direct viral effects. Emerging evidence demonstrates that enteric viruses can interact with the gut microbiome, potentially influencing systemic inflammation and immune responses that affect cardiovascular health. The gut microbiome is essential in modulating inflammation and immune responses, and its interaction with enteroviruses may have implications for CVD pathogenesis [125]. The gut virome contributes to the pathogenesis of CVD primarily through the induction of myocarditis, evasion of the immune response, and manipulation of host cellular processes. The interplay between the gut virome, the immune system, and the gut microbiome highlights the complexity of these interactions and their potential impact on cardiovascular health. Understanding these mechanisms is crucial for developing targeted therapies and preventive strategies against enterovirus-induced CVDs.

### 5.2 Current Challenges

Research on the gut virome has advanced our understanding of its intricacies and significance for human health; however, it encounters substantial limitations that impede comprehensive understanding. A primary obstacle is the considerable genetic variability of intestinal viruses, which complicates their description and categorization. Numerous viruses are unclassified or insufficiently understood, partly because of the lack of adequate reference databases and the prevalence of conserved genes across viruses, frequently termed ‘viral dark matter’ [126, 127]. This variability makes precisely identifying viral species and understanding their roles within the gut ecosystem challenging. Another vital concern pertains to the methodological constraints associated with virome studies. Conventional sequencing techniques, including polymerase chain reaction amplification, may introduce biases that affect the detection of low-abundance viral genomes, leading to an incomplete representation of virome composition [126, 128]. This highlights the need for more advanced sequencing technologies for more precise virome characterization. Furthermore, our current knowledge regarding the functional roles of enteroviruses is limited. Although certain studies have started investigating the interactions between the gut virome and intestinal bacteria, the detailed mechanisms through which the gut virome influences host health and disease are largely unexplored [101, 129]. For instance, the capacity of phages to adjust bacterial populations and, thus, host immune responses is an area that demands additional research to clarify the virome’s effect on health outcomes [130]. Additionally, there are considerable gaps in our comprehension of the interactions between enteroviruses and fungi. The causal relationship between the gut virome and CVD is largely unclear, and clinical studies investigating gut virome-related treatments are limited. Given that the gut virome can vary significantly at different stages of the same disease and among individuals with the same condition [131], it is challenging to ascertain the consistent direction of changes in the gut virome and whether these alterations are causative or consequential in CVD. Additional large-scale human cohort trials and translational animal studies are essential to better evaluate trends and causal relationships. While progress is being made in studying the gut virome, substantial gaps persist. Addressing these challenges is essential for enhancing our understanding of the gut virome and its role in CVD.

### 5.3 Future Directions

Studies on the association between gut virome and CVD are in their early stages; however, their potential impact on cardiovascular health is increasingly recognized, emphasizing significant opportunities for future investigation in this field. As research in this area evolves, several future directions that may enhance our understanding of the gut virome’s impact on cardiovascular health can be identified. One promising avenue is investigating the association between gut virome and gut microbiome, particularly how these interactions affect cardiovascular health. Recent studies have indicated that the gut microbiota can modulate the effects of viruses and *vice versa* [132, 133], indicating a complex interplay that may affect inflammation and metabolic processes related to CVD. Understanding these interactions can lead to new therapeutic strategies that target the virome and microbiome to mitigate cardiovascular risk factors. Another critical direction for future research is identifying and characterizing specific viral species associated with CVD. While some studies have begun to investigate the gut virome in relation to conditions such as AS, there remains a significant gap in knowledge regarding which specific viruses are implicated in cardiovascular diseases [101]. Investigating the presence and abundance of particular phages and their relationships with bacterial hosts could provide insights into their roles in disease mechanisms, including inflammation and lipid metabolism [134]. Moreover, developing advanced metagenomic and metatranscriptomic techniques is essential for a more comprehensive understanding of the gut virome. These technologies can facilitate the identification of viral genomes and their functional roles within the gut ecosystem, allowing researchers to assess how viral infections may affect host metabolism and immune responses related to CVD. Such approaches may aid in understanding the dynamics of viral populations over time and their potential shifts in response to dietary changes or disease states [134, 135]. Additionally, longitudinal studies that track changes in the gut virome in relation to cardiovascular health over time will be essential. These studies can help establish causal relationships between viral dynamics and cardiovascular outcomes, providing a clearer insight into how the virome may affect disease progression or recovery. Such research may identify potential biomarkers for early detection of CVDs based on virome composition [136]. Furthermore, the therapeutic potential of modulating the gut virome offers an exciting direction for future research [137, 138]. Investigating the effects of interventions, including probiotics, prebiotics, or fecal microbiota transplantation, on the gut virome and its subsequent impact on cardiovascular health may lead to innovative treatment strategies [139, 140]. Existing studies have demonstrated that the gut virome exhibits long-term efficacy in treating specific diseases [141], and in some cases, it exhibits greater efficacy than fecal bacterial transplantation [142, 143]. This highlights the significant potential of the gut virome in disease management. Integrating findings from gut virome research with broader epidemiological studies on cardiovascular health could enhance our understanding of the public health implications of gut viruses. Recent studies have demonstrated that high-fat diets [144], alcohol consumption [73, 145], obesity [146], and stress [147] can alter the intestinal microbiota, including gut virome, by impacting host immunity. These factors contribute to the risk of CVD. By correlating virome profiles with lifestyle factors, dietary habits, and genetic predispositions, researchers can better understand the multifactorial nature of CVD and the role of the gut virome within this context.

## 6. Conclusion

This review represents the first systematic exploration of the relationship between the gut virome and CVD, integrating virome-centric evidence into the CVD pathogenesis framework and comprehensively synthesizing the latest advancements in gut virome research within this context. In contrast, previous reviews have primarily focused on associations between the gut bacterial microbiome and CVD. We delineate the unique mechanistic pathways through which gut viruses modulate host physiology—including microbial community dynamics, immune regulation, and metabolic reprogramming. Our analysis integrates cutting-edge findings from viral metagenomics and large-scale clinical cohort studies, establishing an innovative framework that transcends conventional bacteria-centric paradigms. The proposed holistic perspective not only reveals previously underappreciated therapeutic targets within the virome but also pioneers novel research dimensions for CVD intervention strategies. However, current limitations in sample size and methodological heterogeneity necessitate further validation through rigorously designed multicenter studies with extended follow-up periods.In conclusion, future studies on the gut virome and CVD should focus on investigating the complex interactions between viruses and the gut microbiome, identifying specific viral species related to CVD, employing advanced sequencing technologies, conducting longitudinal studies, investigating therapeutic interventions, and integrating findings with broader epidemiological data. These directions can significantly advance our understanding of the role of the gut virome in cardiovascular health and disease, paving the way for new preventive and therapeutic strategies.
